# PHB2 interacts with LC3 and SQSTM1 is required for bile acids-induced mitophagy in cholestatic liver

**DOI:** 10.1038/s41419-017-0228-8

**Published:** 2018-02-07

**Authors:** Yongtao Xiao, Ying Zhou, Ying Lu, Kejun Zhou, Wei Cai

**Affiliations:** 1 Department of Pediatric Surgery, Xin Hua Hospital, School of Medicine, Shanghai Jiao Tong University, Shanghai, China; 2 Shanghai Institute of Pediatric Research, Shanghai, China; 3 Shanghai Key Laboratory of Pediatric Gastroenterology and Nutrition, Shanghai, China

## Abstract

Mitophagy is a major pathway for clearance of injured mitochondria. However, whether mitophagy is involved in the cholestasis-induced damages of hepatic mitochondria remains unknown. We here aimed to investigate the molecular links between cholestasis and hepatic mitophagy. We show that mitophagy is increased significantly in livers of biliary atresia (BA) that is cholestatic disease in infants. The mitochondrial-toxicity bile acids treatment increases the activities of mitophagy in hepatocytes. Mechanistically, we find that the prohibitin 2 (PHB2) is crucial for cholestasis-mediated mitophagy in vitro. On the one hand, PHB2 binds the autophagosomal membrane-associated protein LC3 upon injured mitochondria via an LC3-interaction region domain. On the other hand, PHB2 forms a ternary protein complex with sequestosome 1 (SQSTM1) and LC3, leading to loading of LC3 onto the damaged mitochondria. Altogether, our study suggests that PHB2 is required for cholestasis-induced mitophagy via LC3 onto the injured mitochondria.

## Introduction

Cholestasis-induced liver injury contributes to liver failure in human cholestatic liver diseases. Although underlying cellular basis is partly understood, the key cellular player is the mitochondria^1–4^. Mitochondrial injuries are widespread in cholestatic livers, with elevated levels of the toxic bile acids causing striking changes in mitochondrial structure and function^2,4^, including the appearance of mitochondrial fragments, reduced mitochondrial membrane potential (MMP) and release of pro-apoptotic proteins into the cytosol. Given that mitochondria injuries are critically involved in the induction of cell death, better understanding of the relationship between mitochondrial injuries and cholestasis-induced liver failure is warranted.

Mitophagy is a selective form of macro-autophagy by which eukaryotic cells degrade damaged mitochondria to protect cells against deleterious effects of damaged mitochondria. The previous studies have been demonstrated that mitophagy is dysregulated in several human diseases, including cancer, neurodegeneration, metabolic disorders, muscle atrophy, aging, and inflammation^5,6^. However, whether the mitophagy is involved in cholestasis-induced liver failure is not known. Prohibitin 2 (PHB2) is a highly conserved inner mitochondrial membrane protein that regulates mitochondrial assembly and function^7–9^. In a recent study, Wei et al.^10^ report that PHB2 acts as a receptor for the mitophagic machinery during the mitochondrial degradation. In this study, we first investigated the roles of cholestasis in the hepatic mitophagy. We next assessed the effects of PHB2 in cholestasis-mediated mitophagy. Our findings demonstrate that PHB2 is required for cholestasis-induced mitophagy in which PHB2 brings LC3 to the damaged mitochondria via interaction with SQSTM1and LC3.

## Results

### Mitophagy is significantly increased in cholestatic livers

**Fig. 1:**
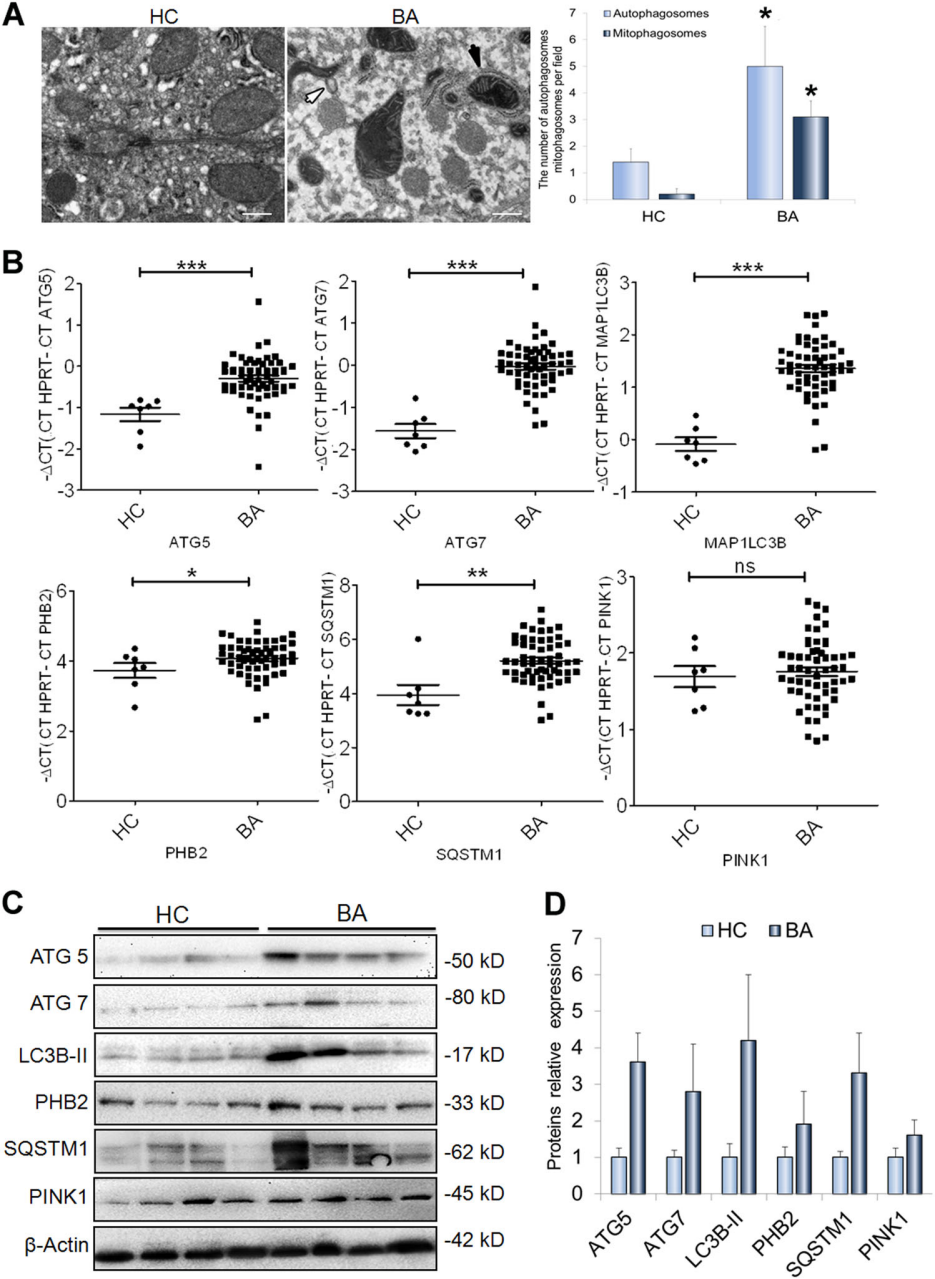
Mitophagy increases in cholestatic livers. **a** Transmission electron microscopy (TEM) analysis of liver sections from biliary atresia (BA) patients (*n* = 8) and controls (*n* = 6) showed mitophagosomes were increased in livers of BA. Black arrows highlight mitophagosomes, white arrows indicate autophagosomes. **b** qRT-PCR analyses were used to determine the levels of MA1LC3B, ATG5, ATG7, PINK1, PHB2, and SQSTM1 mRNAs in liver tissues from BA patients (*n* = 65) and controls (*n* = 7). **c** Western blot analyses of ATG5, ATG7, LC3B, PHB2, SQSTM1, and PHB2 expression in liver tissues from BA patients (*n* = 4) and controls (*n* = 4). **d** Quantification of the protein results in (**c**). HC healthy control, BA biliary atresia; scale bar = 1 μm (**a**), 50 μm (**e**). *p < 0.05, **p < 0.01, ***p < 0.001

As the mitophagy plays an important role in liver diseases^11–14^, we here analysis the roles of mitophagy in the biliary atresia (BA) that is a severe chronic cholestasis disorder of infants. As shown in Fig. 1a, it showed that the mitophagosomes increased significantly in livers of BA patients (*n* = 8) when compared to ones in the control subjects (*n* = 6). (Fig. 1a).The qRT-PCR and western-blot analysis showed that the levels of messenger RNA (mRNA) and protein for mitophagic markers, including ATG5, ATG7, MAP1LC3B, PINK1, and SQSTM1, were evidently up-regulated in the livers of the BA patients related to the control subjects (Fig. 1b–d). Consistently, immunofluorescence staining analysis of the liver sections revealed the proteins LC3B, SQSTM1, and PINK1 expressed specifically in the cytoplasm and significantly elevated in the livers of BA patients compared to those of the controls (Supplementary Figure 1).

### PHB2 is increasingly expressed in injured mitochondria in cholestatic livers

**Fig. 2:**
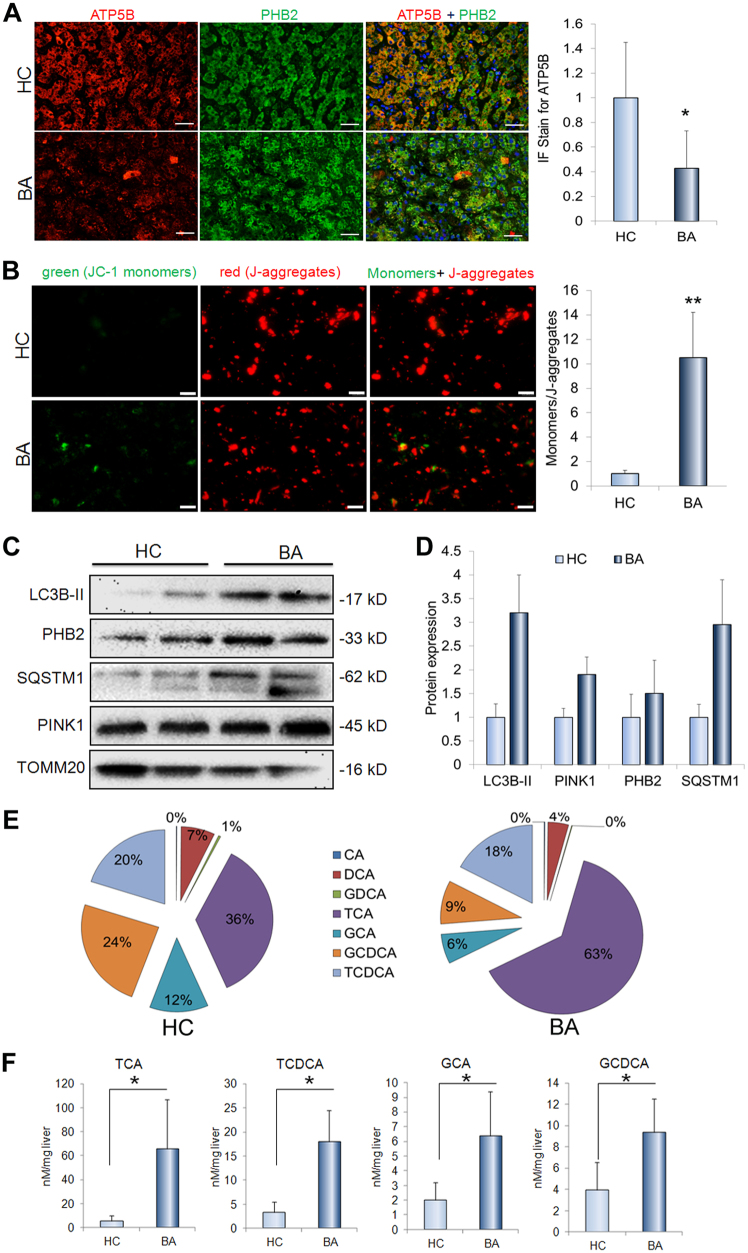
The mitophagy proteins accumulates in mitochondria and mitochondria-targeted bile acids. **a** Representative images and quantification of ATP5B immunofluorescence staining of liver sections from BA patients (*n* = 9) and controls (*n* = 5). **b** MMP analysis for isolated liver mitochondria from BA patients (*n* = 9) compared to controls (*n* = 5). **c** The levels of proteins LC3B, PHB2, SQSTM1, PINK1, and TOMM20 in the isolated liver mitochondria from BA patients (*n* = 2) compared to controls (*n* = 2). **d** Quantification of the protein results in (**c**). **e** The profile of bile acids in the mitochondria from BA patients (*n* = 9) and controls (*n* = 5). **f** The contents of bile acids GCA, TCA, GCDCA, and TCDCA in mitochondria of BA patients (*n* = 9) and controls (*n* = 5). Scale bar = 50 μm (**b**), 10 μm (**c**). *p < 0.05, **p < 0.01

To evaluated whether the mitochondria is injured in cholestatic livers, we measured ATP synthase subunit-β (ATP5B) expression in the livers and assessed the MMP on the isolated hepatic mitochondria. The ATP5B, a subunit of mitochondrial ATP synthase (complex-V) for ATP biosynthesis, plays an important role in maintaining the energy homeostasis in the cells. As shown in Fig. 2a, it showed that ATP5B decreased significantly in livers of the BA patients relative to control subjects (Fig. 2a). The MMP provides a valuable indicator of cells’ health and functional status. The dye JC-1 facilitates discrimination of energized and deenergized mitochondria because normally the green fluorescent monomers form red fluorescent J-aggregates, when concentrated in energized mitochondria in response to their higher membrane potential^15^. We here showed that green fluorescent monomers significantly increased in the isolated hepatic mitochondria from BA patients compared to control subjects (Fig. 2b). Prohibitin 2 (PHB2) is the inner mitochondrial membrane protein^10,16^. We here showed that the expression of PHB2 was significantly increased in the livers of BA patients relative to controls (Fig. 1b–d and Supplementary Figure 1). We also demonstrated that PHB2, as well as mitophagy markers were increasingly expressed or accumulated in the isolated the mitochondria from the liver tissues of BA patients compared to the matched controls (Fig. 2c, d).

### The bile acids targets mitochondria in cholestatic livers

We recently reported that the bile acids GCA, TCA, GCDCA, and TCDCA were abundant in livers, and were greatly higher in livers from BA patients compared to the controls^17^ In this study, we isolated the mitochondria from liver tissues and then analyzed the bile acids profile. As shown in Fig. 2e, it showed that the GCA, TCA, GCDCA, and TCDCA were the main bile acids in the mitochondria (Fig. 2e). In addition, the bile acids GCA, TCA, GCDCA, and TCDCA dramatically elevated in hepatic mitochondria of BA patients related to ones of controls subjects (Fig. 2f). To confirm whether GCA, TCA, GCDCA, and TCDCA that were abundant in mitochondria in cholestatic liver directly bind the hepatic mitochondria, we used isolated mitochondria to incubate with mixture of GCA, TCA, GCDCA, and TCDCA and further to determined their binding ability. It showed that concentration of GCA, TCA, GCDCA, and TCDCA in mitochondria dramatically higher in bile acids-treated group than controls (Supplementary Figure 2A). Given that the TCDCA and GCDCA are toxic to cells, we here to determine whether they are injurious to hepatic mitochondria. We showed that either TCDCA or GCDCA treated-cells had much lower ATP5B expression than the control cells (Supplementary Figure 2B). In addition, treatment of either TCDCA or GCDCA significantly reduced the MMP (Supplementary Figure 2C).

### The mitochondrial-toxicity bile acids induced mitophagy in liver cells

**Fig. 3:**
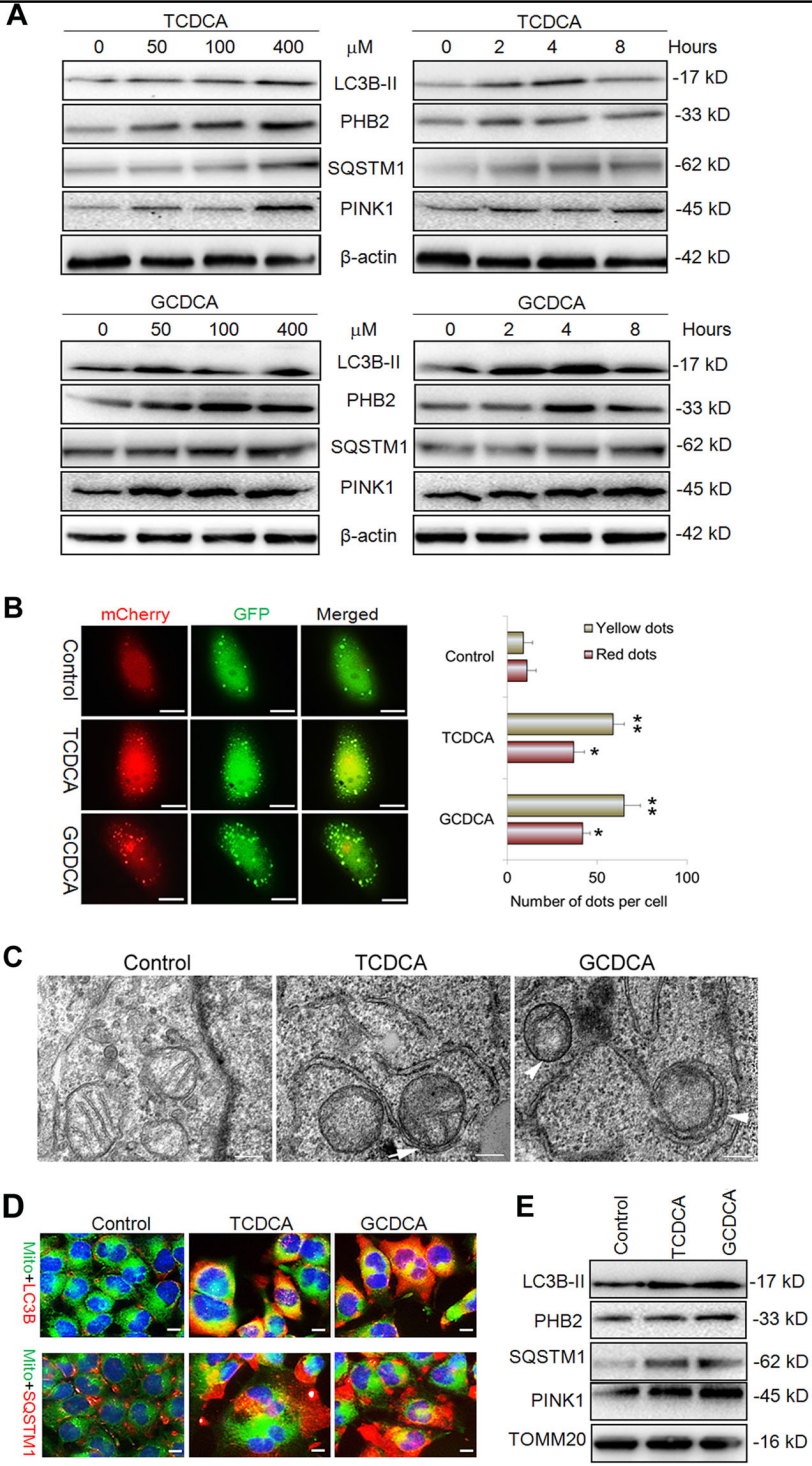
The bile acids induce mitophagy in liver cells. **a** Western blot analysis for the proteins LC3B, PHB2, SQSTM1, PINK1 and actin following treatment of GCDCA or TCDCA. **b** Representative images of L02 cells transfected Ad-mCherry-GFP-LC3B adenovirus after GCDCA or TCDCA treatment (400 μM, 24 h). The number of red and yellow LC3 dots per cell were counted. **c** Representative images of TEM analysis for the L02 cells with TCDCA or GCDCA treatment. **d** Representative images of LC3B and SQSTM1 immunofluorescence staining of L02 cells that treated with GCDCA or TCDCA.** e** The levels of proteins LC3B, PHB2, SQSTM1, PINK1 and TOMM20 in the isolated hepatic mitochondria followed treated with GCDCA or TCDCA. Scale bar = 10 μm (**b**), 5 μm (**c**), 5 μm, *p < 0.05, **p < 0.01

To figure out whether the mitochondrial-toxicity bile acids can induce the mitophagy in liver cells, the different concentration of GCDCA (0–400 μM) and TCDCA (0–400 μM) were used to treat the L02 liver cells for different time. The western blot analysis showed that LC3, PHB2, SQSTM1, and PINK1 increasingly expressed at the indicated time after treatment with GCDCA or TCDCA (Fig. 3a). We further transfected L02 liver cells with Ad-mCherry-GFP-LC3B adenovirus to monitor the autophagy flux in the presence of GCDCA or TCDCA. As shown in Fig. 3b, both yellow and red dots increased significantly after treatment of GCDC or TCDCA. Owing to the fact that yellow dots (merged by mCherry and GFP fluorescence) indicate the autophagosomes that are not fused with lysosome, while red dots (mCherry fluorescense) indicate the compartments that have been fused with lysosome. Thus, the increase of both red and yellow dots indicates the activation of autophagy, suggested that GCDC or TCDCA could induce autophagy in liver cells. In addition, the transmission electron microscopy (TEM) analysis showed that the mitophagosomes appeared in the hepatic cells after treatment with GCDC or TCDCA (Fig. 3c). Furthermore, immunofluorescence staining showed that LC3B and SQSTM1 increasingly accumulated in the mitochondria in the presence of GCDCA or TCDCA (Fig. 3d). To confirm this result, we used western blot to analyze the expression of them in the isolated mitochondria. As shown in Fig. 3e, the levels of protein LC3B, PHB2, SQSTM1, and PINK1 increased evidently in the mitochondria after treatment of GCDC or TCDCA (Fig. 3e).

### PHB2 depletion inhibits bile acids-induced mitophagy in liver cells

**Fig. 4:**
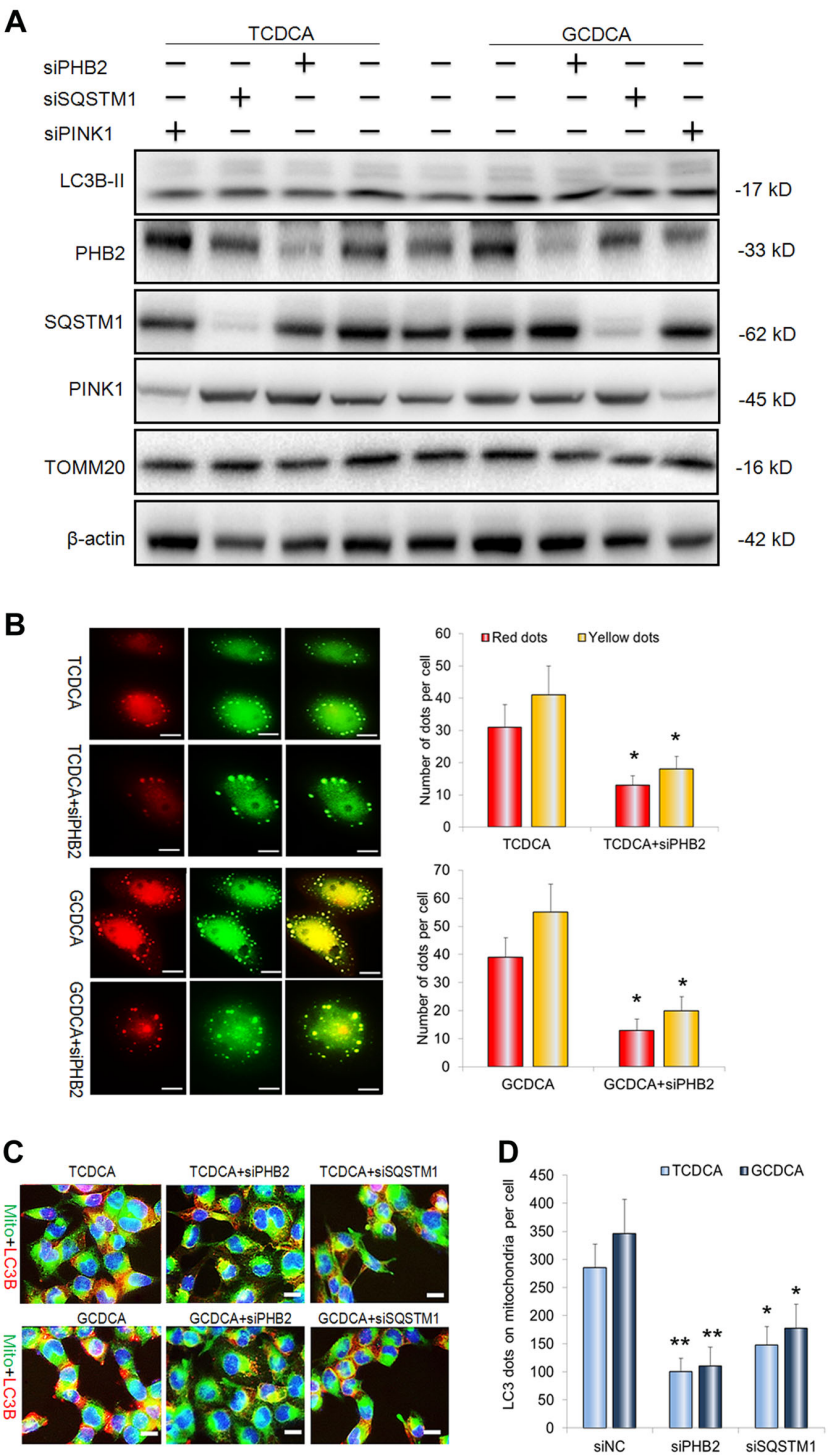
PHB2 depletion inhibits bile acid-indiced mitophagy in liver cells. **a** Western blot analysis for the proteins LC3B, PHB2, SQSTM1, PINK1, and actin in L02 cells after PHB2 siRNA, SQSTM1 siRNA, PINK1 siRNA transfection with treatment of GCDCA and TCDCA. **b** Representative images of L02 cells transfected Ad-mCherry-GFP-LC3B adenovirus in the presence of GCDCA or TCDCA treatment with or without PHB2 siRNA transfection. The number of red and yellow LC3 dots per cell were counted. **c** Representative images of LC3 dots on the mitochondria after GCDCA or TCDCA treatment with or without PHB2 siRNA or SQSTM1 siRNA transfection. **d** The number of red LC3 dots on the mitochondria per cell was counted. Scale bar = 10 μm (**b**), 5 μm (**c**), 5 μm, *p < 0.05, **p < 0.01

To further clarify the mechanism that bile acids-induced mitophagy in liver cells, siRNAs for PHB2, SQSTM1, and PINK1 were transfected 48 h before GCDCA or TCDCA treatment. Western blot analysis indicated that proteins PHB2, SQSTM1, and PINK1 were undetectable after transfected with their corresponding siRNAs (Fig. 4a). For expression of autophagy marker LC3B-II, it decreased but not evidently in the presence of PHB2-, SQSTM1-, and PINK1- knockdown (Fig. 4a). In contrast to, it showed that PHB2 knockdown significantly suppressed the GCDC- or TCDCA-increased red and yellow dots (Fig. 4b). We also found that PHB2 depletion significantly decreased LC3B dots on the mitochondria, suggests PHB2 is essential to recruit LC3B to mitochondria during the mitophagy (Fig. 4c, d). Interestingly, the SQSTM1 knockdown evidently reduced the LC3B dots on the mitochondria (Fig. 4c, d).

### PHB2 interacts with LC3B and SQSTM1 during the bile acids-induced mitophagy

**Fig. 5:**
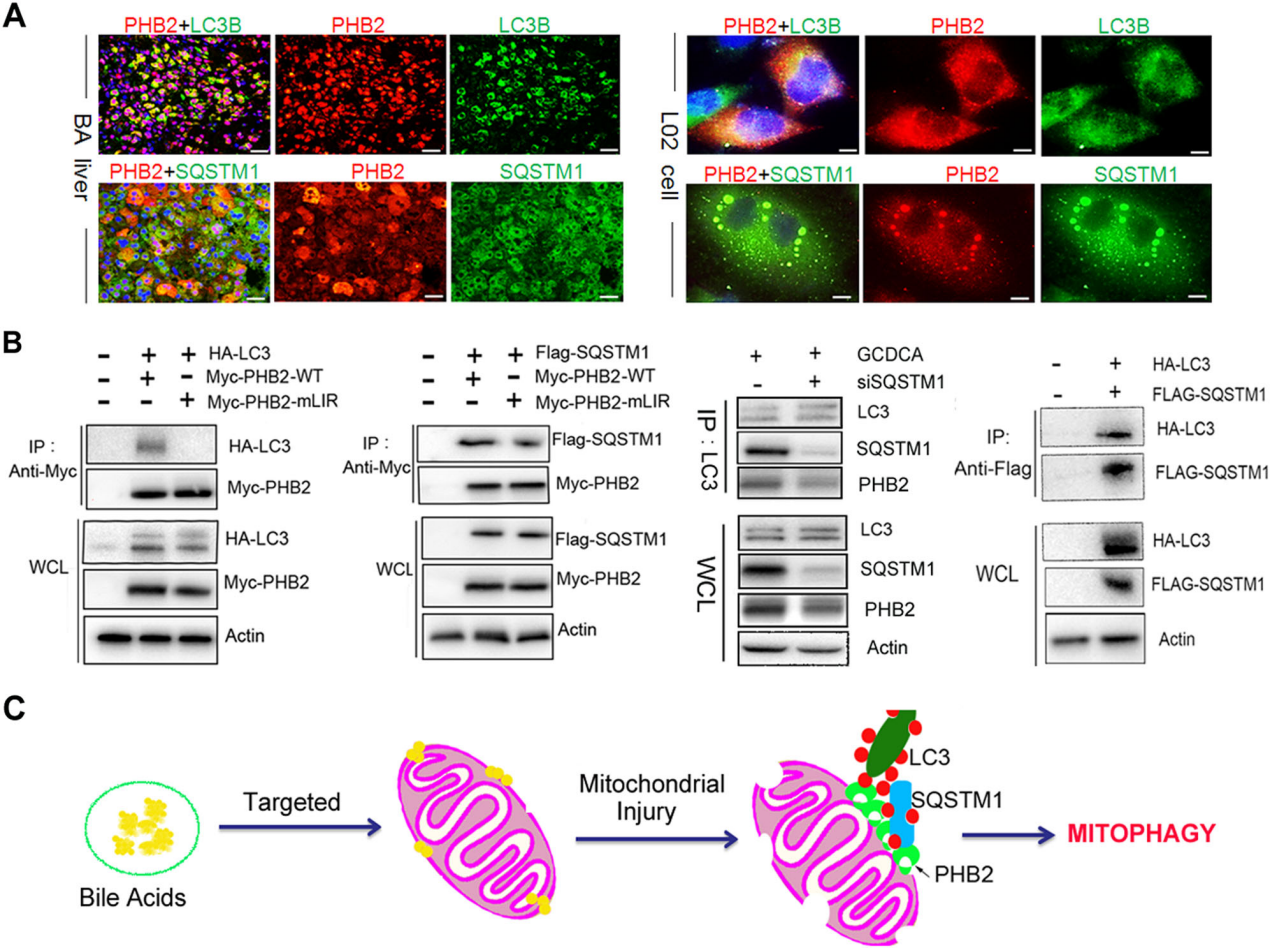
PHB2 binds LC3 and interacts with SQSTM1 during the bile acid-indiced mitophagy. **a** Representative images showing co-localization of PHB2 and LC3B, PHB2, and SQSTM1 in the liver sections of BA patients and L02 liver cells. **b** Co-immunoprecipitation analysis for PHB2 with LC3, as well as SQSTM1 in L02 cells. **c** The scheme illustrates a potential mechanism by which bile acids-induced mitophagy in livers. Scale bar = 25 μm (**a**), 10 μm (**b**), 5 μm

Because PHB2 knockdown resulted of reduced the number of LC3 dots on the mitochondria (Fig. 4c, d), we hypothesized that PHB2 may affect the mitochondria via recruiting LC3 during the mitophagy. As shown in Fig. 5, immunofluorescence analysis showed that PHB2 colocalized with LC3B in the cholestatic livers of BA and GCDCA-treated L02 liver cells (Fig. 5a). Moreover, PHB2 also colocalized with SQSTM1 in the cholestatic livers and GCDCA-treated liver cells (Fig. 5a). Using immunoprecipitation analysis, we observed that PHB2 interacted with LC3B in the transfected cells after treatment of GCDCA (Fig. 5b). Next, we determined the domains responsible for the interaction between PHB2 and LC3 using mutants. A previous study has been identified the LIR domain of PHB2 is essential for mitophagy function^10^. We here showed that the PHB2 LIR domain Y121A/L124A mutant blocked PHB2-Myc immunoprecipitation of LC3 in L02 cells with GADCA-treatment. Using immunoprecipitation analysis, we also observed that PHB2 bound to SQSTM1 in the transfected cells and SQSTM1 knockdown cells (Fig. 5b). Consistent with the fact that SQSTM1 is an important adapter of LC3, we here also showed that SQSTM1 interacted with LC3 in the bile acids-treated cells (Fig. 5b).

## Discussion

In cholestatic livers, the accumulated bile acids are known to induce liver damages via activating apoptotic pathways or death receptors^18^. However, the role of bile acids in hepatic autophagy and mitophagy is not known. In the current study, we first found that bile acids induced the mitochondrial injuries and increased mitophagy in cholestatic livers. We then identified a novel regulatory mechanism that is implicated in cholestasis-induced mitophagy. Our study suggests that the PHB2 is involved in cholestasis-mediated mitophagy via recruitment of the autophagosomal membrane-associated protein LC3 onto the damaged mitochondria.

BA is a typical cholestatic neonatal disease that is a leading liver disease caused death in infants^19,20^. We here found that strong mitochondrial structure alterations and loss of the MMP in the livers of BA patients, suggests the bile acids induce mitochondrial destruction or damages in cholestatic livers. Simultaneously, we observed that autophagosomes and mitophagosomes increased significantly in livers of BA patients compared to control subjects. In addition to, both the mRNA levels and the protein levels of the essential autophagy genes were significantly increased in livers of BA patients. These results suggest that the cholestasis is associated with increased-mitophagy in cholestatic livers. However, the link between cholestasis and mitophagy is unknown. In livers, newly synthesized bile acids were conjugated with either glycine or taurine, generating TCDCA, GCDCA, TCA, and GCA. We recently reported that hepatic bile acids, TCDCA, GCDCA, TCA, and GCA in BA patients were greatly increased compared to the controls^17^. In this study, we showed that TCDCA, GCDCA, TCA, and GCA were the most abundant bile acids in the hepatic mitochondria, and they were increased significantly in the hepatic mitochondria of BA patients compared to control subjects. In vitro, we confirmed that TCDCA, GCDCA, TCA, and GCA could tightly bind to hepatic mitochondria. In line with the established major hepatocellular toxicity of GCDCA and TCDCA in cells and whole-organ models^4^, we here demonstrated that the bile acids GCDCA and TCDCA have toxic potential to liver mitochondria by enhancing mitochondrial permeability.

It is known that mitochondrial dynamics is maintained via mitochondrial fission/fusion and mitophagy^21–24^. If damage accumulates in mitochondria, the mitochondria are aggregated and segregated by fission and followed eliminated by mitophagy. We thus suppose that mitochondrial-toxicity bile acids GCDCA and TCDCA may induce mitophagy in liver cells. Indeed, we indicated that the expression of autophagy related proteins and the autophagy flux increased evidently in liver cells after treating with GCDCA or TCDCA. Moreover, the proteins LC3, SQSTM1, PHB2, and PINK1 increasingly expressed and accumulated in mitochondria. Therefore, we suggest that GCDCA or TCDCA can induce mitochondrial injuries and further increased mitophagy in cholestatic livers. To further clarify the mechanisms for the bile acids-induced mitophagy, we investigated roles and underlie mechanisms of PHB2 in the process of bile acids-mediated mitophagy. PHB2 is the inner mitochondrial membrane protein that regulates mitochondrial assembly and function. In a recently published work, Wei et al.^10^ demonstrate that PHB2 plays an important role in mitophagy via acting as a component for the mitophagic machinery. In this study, investigate whether PHB2 regulates mitophagy in the cholestatic livers. We here observed that PHB2 increased in cholestatic livers and bile acids-treated liver cells. PHB2 knockdown significantly inhibited the GCDCA- or TCDCA-induced mitophagy in liver cells. Using the binding assays, we demonstrated that PHB2 coimmunoprecipitate with LC3. We also identified the LC3-interacting region (LIR) motif of PHB2 and showed that mutations in the critical LIR residues (Y121A/L124A) blocked LC3 binding by PHB2. We also found that PHB2 could directly bind to the SQSTM1 that is an important adapter of LC3^25^. We here observed that SQSTM1 accumulated onto mitochondria during the mitophagy. Knockdown of SQSTM1 expression suppressed bile acids-induced LC3 recruitment into the mitochondria. Thus, except directly binding the LC3B, PHB2 may interact with SQSTM1 to recruit LC3 during bile acids-mediated mitophagy.

In conclusion, we suggest that PHB2 is essential to cholestasis-induced mitophagy in the cholestatic liver via directly binding the LC3 and may interact with SQSTM1 to recruit LC3 into the impaired mitochondria.

## Materials and methods

### Specimens

A total of 65 liver specimens were retrieved from BA patients who underwent surgery. Seven normal adjacent non-tumor tissues that were taken from the hepatoblastoma patients used as controls. All patients’ guardians provided written informed consent. This study was approved by the Faculty of Medicine’s Ethics Committee of Xin Hua hospital (XHEC-C-2016-063). The clinical characteristics of the patients are presented in Supplementary Table 1. All methods in this study were carried out in accordance with the relevant guidelines.

### Transmission Electron Microscopy (TEM)

TEM was performed as described previously^26^. The detal Details of TEM procedures are provided in the Supplementary materials.

### qRT-PCR and western blot

Total RNA was extracted with Trizol reagent (Invitrogen, USA) according to the protocol of the manufacture. A High Capacity cDNA Reverse Transcription kit (Applied Biosystems, Foster City, CA) and a SYBR-Green Universal Master Mix kit (Applied Biosystems, Foster City, CA) were employed to detect the levels of the genes. The primers are listed in Supplementary Table 2. The western blots were performed as previously described^27^. Antibodies to Atg5 (Cell signaling technology, Danvers, MA, USA, dilution, 1: 500), Atg7 (Cell signaling technology, Danvers, MA, USA, dilution, 1: 500), PINK1(Proteintech Group, Chicago, USA, dilution, 1: 200), PHB2 (Proteintech Group, Chicago, USA, dilution, 1: 200), SQSTM1(Proteintech Group, Chicago, USA, dilution, 1: 200), LC3B (Abcam Inc, Cambridge, UK, dilution, 1: 100), and TOMM20 (Proteintech Group, Chicago, USA, dilution, 1: 100) were used here. The detal Details of qRT-PCR and western blot are provided in Supplementary materials.

### Immunofluorescence staining

Antibodies for PINK1(Proteintech Group, Chicago, USA, dilution, 1: 50), PHB2 (Proteintech Group, Chicago, USA, dilution, 1:100), SQSTM1(Proteintech Group, Chicago, USA, dilution, 1: 100), ATP5B (Proteintech Group, Chicago, USA, dilution, 1: 100) and LC3B (Abcam Inc, Cambridge, UK, dilution, 1: 50) were used in this study. Details of IF staining are provided in Supplementary materials.

### Bile acids measurements

Bile acids in the mitochondria and liver were measured according to the previously reported method^28,29^. Details of bile acids analyzed procedures are provided in Supplementary materials.

### Cell culture and transfection

Cells from the normal human liver cell line L02 (shanghai Fuxiang Biotechnology Co., Ltd., China) were cultured in RPMI 1640 Medium supplemented with 10% fetal bovine serum (FBS) at 37 °C with 5% CO_2_ in a humidified atmosphere. Transient transfections with small interfering RNAs (siRNAs) were performed using Lipofectamine® RNAiMAX Transfection Reagent (Thermo Fisher Scientific) following the manufacturer’s protocol. The small interfering RNA (siRNA) duplexes of PHB2, SQSTM1, and PINK1 were synthesized by GenePharma (Shanghai, China). The sequences of siRNAs are listed in Supplementary Table S.

### Autophagy flux analysis

L02 cells were grown on 24-well plates and reached 50–70% confluence at the time of infection. After two washes, cells were infected with Ad-mCherry-GFP-LC3B adenovirus (Beyotime Institute of Biotechnology, Shanghai, China) at a multiplicity of infection of 100 for 24 h. The infected cells treated with either 400 μM GCDCA or 400 μM TCDCA for 16–32 h. Following indicated treatment, autophagy flux was observed under laser scanning confocal microscope (Leica, Wetzlar, Germany). Autophagy flux was evaluated by calculating the number of yellow and red puncta.

### Co-immunoprecipitation assay

For co-immunoprecipitation analysis for protein interaction with PHB2, either wild-type Myc-PHB2 or a Myc-PHB2 LIR domain (Y121A/L124A) mutant was co-transfected with either HA-LC3 or Flag-SQSTM1 into L02 cells for 48 h using Lipofectamine-2000 (Invitrogen). For analying the interaction between SQSTM1 and LC3, HA-LC3, and Flag-SQSTM1co-transfected into L02 cells for 2 days. After treated these cells with 400 μM GCDCA for 4 h, the cells were rinsed with ice-cold PBS and lysed in ice-cold scraped into IP/lysis buffer (Cell Signaling Technology) with a protease inhibitor cocktail (Pierce), and the total supernatant protein was incubated with Myc-Tag (Sepharose Bead Conjugate, Cell Signaling Technology) or Flag-Tag (Sepharose Bead Conjugate, Cell Signaling Technology) with rotation overnight at 4 °C. The immunoprecipitated proteins washed twice with IP/lysis buffer, and was collected by centrifugation at 14,000 × *g* for 10 min. The immunoprecipitated proteins were denatured in SDS sample buffer, boiled for 5 min, and analyzed by western blotting using the HA (Cell Signaling Technology, dilution 1:200), Flag (Cell Signaling Technology, dilution 1:200), Myc (Proteintech Group, 1:100), and appropriate antibodies. Whole cell lysates were prepared and samples were analyzed by western blot with appropriate antibodies.

### Statistical analysis

All data are shown as mean ± SD. Statistical difference was assesed using Student’s unpaired *t*-test (two-tailed) for two group comparison or one-way ANOVA with Tukey’s Multiple Comparison test for more than two groups by GraphPad Prism 5.0 software. *P* < 0.05 was considered as statistically significant.

## Electronic supplementary material

Spplementary material
