# Supplementary material for: PHB2 interacts with LC3 and SQSTM1 is required for bile acids-induced mitophagy in cholestatic liver
Source: Cell Death Dis. 2018 Feb 7;9(2):160. doi: 10.1038/s41419-017-0228-8 (PMC5833850; doi:10.1038/s41419-017-0228-8)
Supplement: Supplementary file 1 — Spplementary material [file 41419_2017_228_MOESM1_ESM.doc]

**Supplemental Information**

**PHB2 Interacts with LC3 and SQSTM1 Is Required for Bile Acids-Induced Mitophagy in Cholestatic Liver**

**Yongtao Xiao, Ying Zhou, Ying Lu, Kejun Zhou, Wei Cai**

**Figure S1, Related to Figure 1**

**
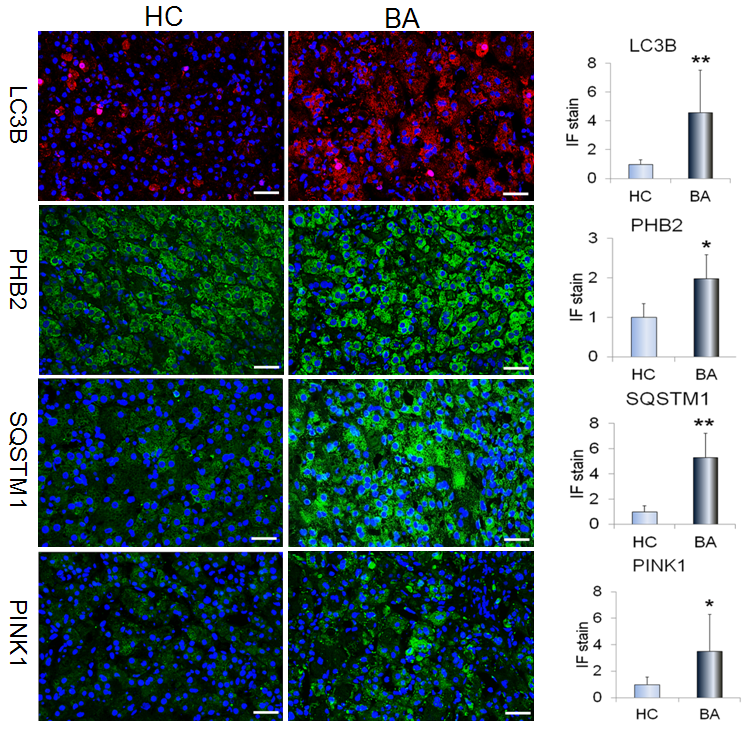
**

**Figure S2, Related to Figure 2**

**
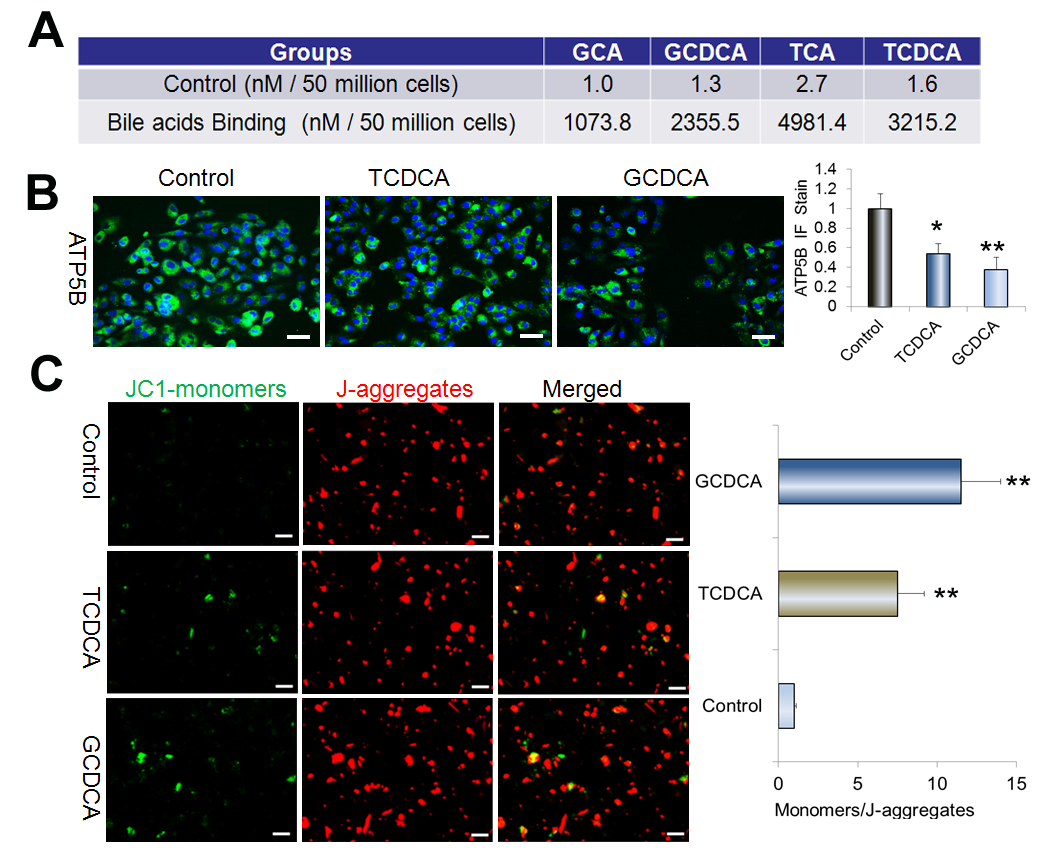
**

**Supplemental Figure Legends**

**Figure S1, Related to Figure 1. Immunofluorescence staining and quantification for LC3B, PHB2, SQSTM1 and PHB2 in liver sections from biliary atresia patients (n=9) and controls (n=5).** HC, healthy control; BA, biliary atresia; Scale bar=1 μm (A), 50 μm (E). *p <0.05, ** p <0.01, *** p <0.001

**Figure S2, Related to Figure 2. The bile acids target mitochondria and induce mitochondria injury in liver cells.**

(A) The concentration of bile acids GCA, TCA, GCDCA and TCDCA in isolated mitochondria after incubation with these bile acids.

(B) Representative images of ATP5B immunofluorescence staining of L02 liver cells that treated with GCDCA and TCDCA.

(C) Measurements of mitochondrial membrane potential (MMP) in isolated hepatic mitochondria after stimulation with GCDCA and TCDCA.

Scale bar=50 μm (B), 10 μm (C), 5 μm (C). *p <0.05, ** p <0.01

**Table S1, Related to Experimental Procedures. The information of patients with biliary atresia and controls**

| **NO** | **Gender** | **Age** | **TBA (μM)** | **ALT**  **(U/L)** | **AST**  **(U/L)** | **γGT**  **(U/L)** | **T-Bilirubin**  **(μM)** | **D-Bilirubin**  **(μM)** |
| --- | --- | --- | --- | --- | --- | --- | --- | --- |
| BA 1 | Female | 2 Years | 89 | 103 | 147 | 238 | 143 | 104 |
| BA 2 | Female | 58 Days | 104 | 318 | 409 | 732 | 202 | 142 |
| BA 3 | Male | 2 Months | 81 | 122 | 166 | 560 | 143 | 99 |
| BA 4 | Female | 8 Days | 46 | 122 | 166 | 290 | 65 | 32 |
| BA 5 | Male | 3 Months | 151 | 135 | 137 | 989 | 68 | 53 |
| BA 6 | Male | 2 Months | 184 | 206 | 435 | 1092 | 197 | 116 |
| BA 7 | Female | 5 Months | 158 | 410 | 406 | 689 | 240 | 190 |
| BA 8 | Male | 39 Days | 125 | 66 | 119 | 832 | 108 | 91 |
| BA 9 | Male | 2 Months | 104 | 261 | 274 | 530 | 123 | 101 |
| BA 10 | Female | 2 Months | 185 | 115 | 157 | 249 | 224 | 151 |
| BA 11 | Male | 2 Months | 164 | 82 | 102 | 267 | 123 | 92 |
| BA 12 | Female | 2 Months | 89 | 99 | 155 | 402 | 195 | 144 |
| BA 13 | Male | 46 Days | 61 | 177 | 150 | 39 | 185 | 143 |
| BA 14 | Male | 90 Days | 97 | 203 | 297 | 160 | 261 | 182 |
| BA 15 | Female | 2 Years | 150 | 129 | 198 | 887 | 146 | 102 |
| BA 16 | Female | 3 Months | 261 | 306 | 336 | 1126 | 136 | 180 |
| BA 17 | Male | 3 Months | 46 | 104 | 118 | 419 | 28 | 21 |
| BA 18 | Female | 3 Months | 66 | 147 | 384 | 447 | 197 | 121 |
| BA 19 | Male | 3 Months | 138 | 103 | 136 | 561 | 162 | 121 |
| BA 20 | Male | 4 Months | 101 | 270 | 262 | 443 | 163 | 75 |
| BA 21 | Male | 5 Months | 167 | 66 | 302 | 784 | 192 | 153 |
| BA 22 | Male | 3 Months | 134 | 168 | 197 | 625 | 309 | 218 |
| BA 23 | Male | 2 Months | 66 | 64 | 93 | 219 | 144 | 119 |
| BA 24 | Male | 3 Months | 68 | 35 | 91 | 216 | 177 | 130 |
| BA 25 | Male | 68 Days | 57 | 77 | 68 | 671 | 109 | 90 |
| BA 26 | Male | 2 Months | 98 | 109 | 126 | 1156 | 136 | 103 |
| BA 27 | Male | 3 Months | 160 | 130 | 141 | 224 | 268 | 192 |
| BA 28 | Male | 50 Days | 141 | 160 | 428 | 288 | 199 | 153 |
| BA 29 | Female | 75 Days | 152 | 258 | 236 | 649 | 217 | 168 |
| BA 30 | Male | 4 Months | 187 | 183 | 384 | 66 | 409 | 266 |
| BA 31 | Female | 2 Months | 165 | 98 | 142 | 764 | 116 | 89 |
| BA 32 | Female | 2 Months | 101 | 157 | 148 | 534 | 150 | 80 |
| BA 33 | Male | 2 Months | 124 | 142 | 224 | 607 | 141 | 110 |
| BA 34 | Male | 31 Days | 132 | 115 | 179 | 387 | 209 | 172 |
| BA 35 | Male | 2 Months | 110 | 101 | 166 | 560 | 143 | 99 |
| BA 36 | Female | 1 Month | 146 | 120 | 169 | 290 | 165 | 132 |
| BA 37 | Male | 2 Months | 122 | 109 | 127 | 589 | 108 | 153 |
| BA 38 | Male | 2 Months | 180 | 229 | 405 | 462 | 177 | 106 |
| BA 39 | Female | 3Months | 133 | 210 | 406 | 689 | 210 | 100 |
| BA 40 | Male | 2 Months | 178 | 166 | 89 | 632 | 228 | 98 |
| BA 41 | Male | 2 Months | 129 | 228 | 275 | 596 | 129 | 107 |
| BA 42 | Female | 2 Months | 185 | 115 | 157 | 249 | 224 | 151 |
| BA 43 | Male | 4 Months | 164 | 82 | 102 | 267 | 123 | 92 |
| BA 44 | Female | 2 Months | 89 | 99 | 155 | 402 | 195 | 144 |
| BA 45 | Male | 2 Months | 161 | 170 | 158 | 89 | 168 | 115 |
| BA 46 | Male | 3 Months | 97 | 203 | 297 | 160 | 261 | 182 |
| BA 47 | Female | 2 Years | 123 | 189 | 498 | 807 | 147 | 100 |
| BA 48 | Female | 3 Months | 121 | 183 | 102 | 193 | 176 | 108 |
| BA 49 | Male | 4 Months | 96 | 154 | 258 | 819 | 128 | 111 |
| BA 50 | Female | 3 Months | 166 | 347 | 484 | 907 | 120 | 147 |
| BA 51 | Male | 1 Months | 138 | 103 | 136 | 561 | 162 | 121 |
| BA 52 | Male | 4 Months | 91 | 170 | 232 | 543 | 263 | 77 |
| BA 53 | Male | 5 Months | 157 | 69 | 325 | 704 | 182 | 143 |
| BA 54 | Male | 2Months | 174 | 268 | 297 | 725 | 335 | 207 |
| BA 55 | Male | 2 Months | 166 | 164 | 193 | 419 | 174 | 159 |
| BA 56 | Male | 3 Months | 168 | 135 | 191 | 316 | 137 | 170 |
| BA 57 | Male | 4Months | 157 | 177 | 468 | 371 | 209 | 190 |
| BA 58 | Male | 2 Months | 198 | 209 | 256 | 856 | 236 | 173 |
| BA 59 | Male | 3 Months | 190 | 150 | 145 | 204 | 208 | 122 |
| BA 60 | Male | 2 Months | 101 | 166 | 328 | 228 | 194 | 157 |
| BA 61 | Female | 1 Months | 112 | 211 | 233 | 849 | 217 | 122 |
| BA 62 | Male | 4 Months | 187 | 183 | 384 | 266 | 709 | 166 |
| BA 63 | Female | 2 Months | 125 | 258 | 342 | 764 | 187 | 83 |
| BA 64 | Female | 2 Months | 103 | 158 | 188 | 654 | 150 | 88 |
| BA 65 | Female | 2 Months | 109 | 198 | 197 | 534 | 274 | 180 |
| Control 1 | Male | 3 Months | 19 | 32 | 70 | 110 | 18 | 5.0 |
| Control 2 | Male | 5 Months | 21 | 20 | 47 | 110 | 4.6 | 1.9 |
| Control 3 | Male | 6 Months | 6.9 | 29 | 49 | 76 | 4.7 | 1.7 |
| Control 4 | Male | 3 Years | 8.7 | 112 | 111 | 225 | 11.5 | 4.7 |
| Control 5 | Female | 10 Months | 20 | 176 | 160 | 352 | 10.3 | 8.8 |
| Control 6 | Male | 9 Months | 3.9 | 126 | 121 | 21 | 12.4 | 4.3 |
| Control 6 | Male | 11 Months | 28 | 85 | 29 | 21 | 5.4 | 3.3 |
| Control 7 | Male | 14 Months | 28 | 85 | 29 | 21 | 5.4 | 3.3 |

TBA, total bile acid (0-10 μM); ALT, alanine aminotransferase (0-75 U/L);AST, aspartate aminotransferase (8-38 U/L); γGT ,γ-glutamyltranspeptidase (16-73 U/L); T-Bilirubin, total bilirubin (3.42-20.52 μM); D-Bilirubin, direct bilirubin (0-6.8)

**Table S2, Related to Experimental Procedures. The sequence of primers and siRNAs**

| **Primers** |  |  |
| --- | --- | --- |
|  | Reverse（5'-3'） | CCTGGCGTCGTGATTAGTGAT |
| HRPT | Forward （5'-3'） | AGACGTTCAGTCCTGTCCATAA |
|  | Reverse（5'-3'） | TGGGACTCCTGGGAATACTG |
| MAP1LC3B | Forward （5'-3'） | AAGGCTTTCAGAGAGACCCTG |
|  | Reverse（5'-3'） | CCGTTTACCCTGCGTTTGTG |
| SQSTM1 | Forward （5'-3'） | CCGTGAAGGCCTACCTTCTG |
|  | Reverse（5'-3'） | TCCTCGTCACTGGAAAAGGC |
| PINK1 | Forward （5'-3'） | CAAGAGGCTCAGCTACCTGCAC |
|  | Reverse（5'-3'） | TGTCTCACGTCTGGAGGCACT |
| ATG5 | Forward （5'-3'） | TGCAGATGGACAGTTGCACA |
|  | Reverse（5'-3'） | CCACTGCAGAGGTGTTTCCA |
| ATG7 | Forward （5'-3'） | AGCGGCGGCAAGAAATAATG |
|  | Reverse（5'-3'） | AACCCAACATCCAAGGCACT |
| PHB2 | Forward （5'-3'） | CGGGCCCAATTCTTGGTAGA |
|  | Reverse（5'-3'） | TCTGGGCTGCTCGAATCTTG |
| PHB2 siRNAs |  |  |
| siRNA-1 | sense（5'-3'） | AGAUUCGAGCAGCCCAGAAUAUCTC |
|  | antisense（5'-3'） | GUUCUAAGCUCGUCGGGUCUUAUAGAG |
| siRNA-2 | sense（5'-3'） | AGAUAAACACCAACCCAGGAAUUCT |
|  | antisense（5'-3'） | CCUCUAUUUGUGGUUGGGUCCUUAAGA |
| SQSTM1 siRNAs |  |  |
| siRNA-1 | sense（5'-3'） | GAUCUGCGAUGGCUGCAAUTT |
|  | antisense（5'-3'） | AUUGCAGCCAUCGCAGAUCTT |
| siRNA-2 | sense（5'-3'） | CCUACGUGAAGGAUGACAUTT |
|  | antisense（5'-3'） | AUGUCAUCCUUCACGUAGGTT |
| siRNA-3 | sense（5'-3'） | CCAGACUACGACUUGUGUATT |
|  | antisense（5'-3'） | UACACAAGUCGUAGUCUGGTT |
| PINK1 siRNAs |  |  |
| siRNA-1 | sense（5'-3'） | GCCAGUACCUUUGUGUGAATT |
|  | antisense（5'-3'） | UUCACACAAAGGUACUGGCTT |
| siRNA-2 | sense（5'-3'） | CCGCAAAUGUGCUUCAUCUTT |
|  | antisense（5'-3'） | AGAUGAAGCACAUUUGCGGTT |
| siRNA-3 | sense（5'-3'） | GAGAAGUGUUGUGUGGAAATT |
|  | antisense（5'-3'） | UUUCCACACAACACUUCUCTT |

**Supplemental Experimental Procedures**

**Transmission electron microscopy (TEM) examination**

The liver samples (1 mm Х 1 mm Х 1 mm) from patients were fixed with 3% glutaraldehyde at room temperature. The tissues were then washed and postfixed with 1% osmium tetroxide in 0.05 mol/L sodium cacodylate buffer (pH 7.4) at 4 °C for 2 hours and stained with saturated uranyl acetate for 3.5 hours at room temperature, dehydrated in graded alcohol and embedded in Eponate 12 resin (Ted Pella, Inc., United States). Sections were then cut with a diamond knife, stained with a saturated solution of uranyl acetate in 50% ethanol and lead citrate. Sections were examined and photographed with a Philips CM120 electron microscope at 80 KV by an experienced electron microscopsit.

**Immunofluorescence staining**

For liver tissues, the liver sections were incubated with xylol and descending concentrations of ethanol. Endogenous peroxidases were removed with 0.3% H2O2. The antibodies of PINK1(Proteintech Group, Chicago, USA, dilution, 1: 50), PHB2 (Proteintech Group, Chicago, USA, dilution, 1: 100), SQSTM1(Proteintech Group, Chicago, USA, dilution, 1: 100), ATP5B (Proteintech Group, Chicago, USA, dilution, 1: 100) and LC3B (Abcam Inc, Cambridge, UK, dilution, 1: 50) were then applied at an optimal concentration overnight in a wet chamber after blocking with 5% bovine serum albumin (BSA) for 1 hour at room temperature. The slides were rinsed in phosphate-buffered saline and incubated with the s anti-mouse IgG conjugated Alexa Fluor® 555 (Cell Signaling Technology, Inc., Danvers, MA, USA) and Anti-rabbit conjugated Alexa Fluor® 488 (Cell Signaling Technology, Inc., Danvers, MA, USA). The slides were rinsed in phosphate-buffered saline (PBS) and counterstained with counterstained with 4’, 6-diamidino-2-phenylindole (DAPI, Sigma, St. Louis, USA). For cellular immunofluorescence, the cells were initially fixed with 4% paraformaldehyde for 10 minutes. The cells were then washed with PBS twice and blocked with 3% BSA contained 0.1% Trion X-100. The cells were incubated with antibodies of LC3B LC3B (Abcam Inc, Cambridge, UK, dilution, 1: 100) and SQSTM1 SQSTM1 (Proteintech Group, Chicago, USA, dilution, 1: 100), at room temperature for 2 hours. After washing three times with PBS, the secondary antibodies conjugated to Alexa Fluor® 555 were applied to these cells. The mitochondria were stained with Mitobright Green (Dojindo Laboratories - Kumamoto, Japan). The images of IF were analyzed using Image Pro Plus software (Media Cybernetics, Rockville, MD, USA).

**Quantitative real-time polymerase chain reaction (qRT-PCR) and western blot**

The liver samples were homogenized using MagNA Lyser Instrument and MagNA Lyser Green Beads (Manassas, VA, USA). Total RNA was extracted with Trizol according to the protocol of the manufacture (Invitrogen, Foster, CA). A SYBR-Green Universal Master Mix kit and a High Capacity cDNA Reverse Transcription kit were employed to detect the levels of the genes. The primers are listed in Supplementary Table 2. For Western blot, the equal amounts of proteins (Liver tissues lysis, 200-400 μg/well; Cell lysis, 20-40 μg/well) were separated by 10% SDS-PAGE, and transferred to nitrocellulose membranes. The membranes were incubated overnight at 4 °C with primary antibodies. Antibodies for Antibodies to PINK1(Proteintech Group, Chicago, USA, dilution, 1: 200), PHB2 (Proteintech Group, Chicago, USA, dilution, 1: 200), SQSTM1(Proteintech Group, Chicago, USA, dilution, 1: 200), LC3B (Abcam Inc, Cambridge, UK, dilution, 1: 100) and TOMM20 (Proteintech Group, Chicago, USA, dilution, 1: 100) were analysed. The membranes were washed with PBS (containing 0.1% Tween) and incubated with horseradish-peroxidase conjugated detected the antigen-antibody complexes using an ECL Plus chemiluminescence reagent kit (Pierce, Rockford, IL, USA).

**Bile Acids Measurements**

Bile acids in the plasma and liver were measured according to the previously reported method . The detection was performed with a Waters ACQUITY ultra performance liquid chromatography (BEH C18 1.7 μm 2.1×100mm column) coupled with Waters Xevo TQ-S triple quadrupole mass spectrometry. Data acquisition and bile acids quantification were performed using the MassLynx 4.1 software (Waters). The bile acid inculded the cholic acid (CA), glycocholic acid (GCA), taurocholic acid ( TCA), chenodeoxycholic acid (CDCA), glycochenodeoxycholic acid (GCDCA), taurochenodeoxycholic acid (TCDCA), deoxycholic acid (DCA), glycodeoxycholic acid (GDCA), taurodeoxycholic acid (TDCA), ursodeoxycholic acid (UDCA), glycoursodeoxycholic acid (GUDCA), tauroursodeoxycholic acid (TUDCA), lithocholic acid (LCA), glycolithocholic acid (GLCA), taurolithocholic acid ( TLCA), hyocholic acid (HCA), glycohyocholic acid (GHCA), taurohyocholic acid (THCA), α-muricholic acid (αMCA), tauro-α-muricholic acid (TαMCA), β-muricholic acid (βMCA), tauro- β-muricholic acid ( TβMCA), ω-muricholic acid (ωMCA), tauro-ω-muricholic acid (TωMCA), hyodeoxycholic acid (HDCA), glycohyodeoxycholic acid (GHDCA), taurohyodeoxycholi acid (THDCA), murocholic acid (MuroCA), dehydrocholic acid (DHCA), glycodehydrocholic acid (GDHCA), taurodehydrocholic acid (TDHCA), 3-dehydrocholic acid (3-DHCA), 7-dehydrocholic acid (7-DHCA), isodeoxycholic acid (isoDCA), apocholic acid (apoCA), 6-ketolithocholic acid (6-KLCA), 7-ketolithocholic acid (7-KLCA), 12-ketolithocholic acid (12-KLCA), 23-nordeoxycholic acid (23norDCA), dehydrolithocholic acid (DHLCA). Deuterated internal standards (IS) lithocholic acid-2,2,4,4-D4 (LCA-D4) and cholic acid-2,2,4,4-D4 (CA-D4) and Chenodeoxycholic Acid 24-Acyl-β-D-glucuronide (CDCA-24G).

**Mitochondrial membrane potential measurement and bile acids targeting mitochondria**

The mitochondrion was isolated from liver tissues and liver cells using Mitochondria Isolation Kit (Beyotime Institute of Biotechnology, Shanghai, China) according to the protocol of the manufacture. For liver tissues, the livers samples were homogenized using Tenbroeck Tissue Grinders (Wheaton) firstly. To assess the mitochondrial membrane potentials, the isolated mitochondria were incubated with 10 mg/mL of JC-1 dye (Invitrogen) for 10 minutes and analyzed using Nikon Eclipse Ti microscope. To determine the ability of bile acids binding mitochondria, the isolated mitochondria were incubated with bile acids mixture contained GCA, TCA, GCDCA, and TCDCA at concentration of 400 μM for 10 minutes, and washed mitochondria twice for bile acids measurement.

**Supplementary Reference**

1. Xie G, Zhong W, Li H, Li Q, Qiu Y, Zheng X*, et al.* Alteration of bile acid metabolism in the rat induced by chronic ethanol consumption. *FASEB J* 2013, **27**(9)**:** 3583-3593.

2. Garcia-Canaveras JC, Donato MT, Castell JV, Lahoz A. Targeted profiling of circulating and hepatic bile acids in human, mouse, and rat using a UPLC-MRM-MS-validated method. *J Lipid Res* 2012, **53**(10)**:** 2231-2241.
